# Design and dosimetric characterization of a transportable proton minibeam collimation system

**DOI:** 10.3389/fonc.2024.1473625

**Published:** 2024-12-17

**Authors:** Mabroor Ahmed, Elke Beyreuther, Sebastian Gantz, Felix Horst, Juergen Meyer, Jörg Pawelke, Thomas E. Schmid, Jessica Stolz, Jan J. Wilkens, Stefan Bartzsch

**Affiliations:** ^1^ Institute of Radiation Medicine (IRM), Helmholtz Zentrum München GmbH, German Research Center for Environmental Health, Neuherberg, Germany; ^2^ Department of Radiation Oncology, TUM School of Medicine and Health, Klinikum rechts der Isar, Technical University of Munich, Munich, Germany; ^3^ Institute of Radiation Physics, Helmholtz Zentrum Dresden-Rossendorf, Dresden, Germany; ^4^ OncoRay – National Center for Radiation Research in Oncology, Faculty of Medicine and University Hospital Carl Gustav Carus, Technische Universitat Dresden, Helmholtz-Zentrum Dresden – Rossendorf, Dresden, Germany; ^5^ Institute of Radiooncology - OncoRay, Helmholtz Zentrum Dresden - Rossendorf, Dresden, Germany; ^6^ Department of Radiation Oncology, Fred Hutchinson Cancer Center, University of Washington, Seattle, WA, United States

**Keywords:** spatially fractionated radiation therapy, proton minibeam radiation therapy, proton minibeam collimation, proton minibeam dosimetry, micro diamond, monte carlo

## Abstract

**Background:**

Proton Minibeam Radiation Therapy has shown to widen the therapeutic window compared to conventional radiation treatment in pre-clinical studies. The underlying biological mechanisms, however, require more research.

**Purpose:**

The purpose of this study was to develop and characterize a mechanical collimation setup capable of producing 250µm wide proton minibeams with a center-to-center distance of 1000µm.

**Methods:**

To find the optimal arrangement Monte Carlo simulations were employed using the Geant4 toolkit TOPAS to maximize key parameters such as the peak-to-valley dose ratio (PVDR) and the valley dose rate. The experimental characterization of the optimized setup was carried out with film dosimetry at the University Proton Therapy beamline in Dresden and the proton beamline of the University of Washington Medical Center in Seattle with 150MeV and 50.5MeV, respectively. A microDiamond detector (PTW, Freiburg, Germany) was utilized at both beamlines for online proton minibeam dosimetry.

**Results:**

A PVDR of 10 was achieved in Dresden and a PVDR of 14 in Seattle. Dosimetry measurements were carried out with EBT3 films at a depth of 5mm in a polymethylmethacrylate (PMMA) phantom. When comparing film dosimetry with the microDiamond, excellent agreement was observed in the valleys. However, the peak dose showed a discrepancy of approximately 10% in the 150MeV beam and 20% in the 50.5MeV beam between film and microDiamond.

**Discussion:**

The characteristics of the minibeams generated with our system compares well with those of other collimated minibeams despite being smaller. The deviations of microDiamond measurements from film readings might be subject to the diamond detector responding differently in the peak and valley regions. Applying previously reported correction factors aligns the dose profile measured by the microDiamond with the profile acquired with EBT3 films in Dresden.

**Conclusion:**

The novel proton minibeam system can be operated independently of specific beamlines. It can be transported easily and hence used for inter-institutional comparative studies. The quality of the minibeams allows us to perform *in vitro* and *in vivo* experiments in the future. The microDiamond was demonstrated to have great potential for online dosimetry for proton minibeams, yet requires more research to explain the observed discrepancies.

## 1 Introduction

Spatially fractionated radiation therapy (SFRT) was first introduced in the early 20th century by Alban Köhler (1), who demonstrated reduced toxicity to the skin when irradiated with a heterogeneous dose pattern. This pattern, which leads to high-dose peaks and low-dose valleys, can be characterized by parameters such as the beam width, the center-to-center (ctc) distance, and the peak-to-valley dose ratio (PVDR). This concept of spatially fractionated dose delivery was revisited in the early 1990s and has since been further developed, leading to different SFRT regimens that are primarily differentiated by their beam width. From these regimens, Minibeam Radiation Therapy (MBRT) and Microbeam Radiation Therapy (MRT) are of main interest in current pre-clinical research (2). MBRT utilizes beam widths of several 100µm, whereas the beam width of MRT is typically below 100µm (3). While MRT is predominantly studied using photon radiation generated by 3rd generation synchrotrons (4, 5), the focus for MBRT has shifted towards particle radiation, with the aim to enhance therapeutic outcomes by leveraging the advantageous characteristics of particles in conjunction with spatial fractionation. The most prominent example is proton Minibeam Radiation Therapy (pMBRT), which can be designed to have spatial modulation in the entrance region and homogeneous tumor coverage due to significant broadening of individual beams in the target (6–8). A wide range of studies have demonstrated that pMBRT is better tolerated by normal tissue while simultaneously offering effective tumor control (7, 9–13). Despite these findings, the underlying biological mechanisms are not well understood. On top of that, an unequivocal set of physical parameters that would optimize the therapeutic outcome of MBRT or MRT has not yet been identified since different groups use different minibeam geometries. To identify the optimal set of parameters, it is essential to ensure the reproducibility of the experimental findings even at different facilities.

When it comes to producing minibeams, there are primarily two approaches. One method is harnessing the charge of protons and using magnets to focus and shape them into a minibeam pattern. This method holds great promise as the quality of the minibeam field is not hindered by scattered particles, which could otherwise compromise dose modulation (14). However, implementing this method requires substantial technological developments (6). The other, more straightforward approach to produce minibeams is using mechanical collimation. The collimators are typically composed of thick metal blocks with slits to allow protons to pass only through specific areas. Prior studies have identified brass as the optimal material for collimating protons into minibeams (15) and other key insights, such as increasing PVDR of the minibeam field with higher thickness of the collimator (16). However, most previously developed collimators for proton minibeam generation were primarily designed for research involving larger animals, such as rats (9, 11, 17). In this study, we aim to extend the applicability of pMBRT research to smaller animals, like mice. Due to their reduced body size, a collimation system capable of producing narrower minibeams with a smaller ctc spacing is essential. We targeted a minibeam width of 250µm with a ctc of 1000µm. Additionally, the minibeam system needed to meet minimal dose modulation requirements, as the PVDR is a key factor for the effectiveness of pMBRT (18). Here, we aimed for a PVDR value of at least 10 to ensure comparability with other configurations (19–21). To accomplish these objectives, we performed Monte Carlo simulations to identify the optimal arrangement of all system components. Subsequently, we established a dosimetric protocol and evaluated the system at two distinct proton facilities, demonstrating that the flexibility of our setup allows for straightforward integration across various beamlines. This feature of our minibeam system is important as it allows inter-institutional comparative studies.

## 2 Materias and methods

### 2.1 Proton facilities

#### 2.1.1 University Proton Therapy Dresden

The experimental hall of the University Proton Therapy Dresden, Germany is equipped with a horizontal research beamline that delivers static pencil proton beams with a maximum energy of 230MeV and an energy-dependent maximal current of 100nA (22). This beamline was operated at a proton energy of 150MeV for our proton minibeam setup. Further, the beamline incorporates a monitor chamber that registers the beam passing through it and quantifies it in terms of monitor units (MU), which can be used to control the dose delivery.

#### 2.1.2 University of Washington Medical Center

The University of Washington Medical Center in Seattle, USA, operates a Scanditronix MC50 multi-particle variable energy cyclotron for fast neutron therapy capable of producing a 50.5MeV proton beam at a maximum beam current of 75µA, which is used for both patient treatment and research purposes. For research applications, the beam can be directed to a separate room (23), where the minibeam setup was installed. A microDiamond detector (Type: 60019, PTW, Freiburg, Germany) was positioned near the beam exit window, providing a measure of the beam flux by registering the dose from scattered particles. This detector was read out by a Keithley 6517B electrometer and used to control the beam delivery.

### 2.2 Development of a proton minibeam setup

**Figure 1:**
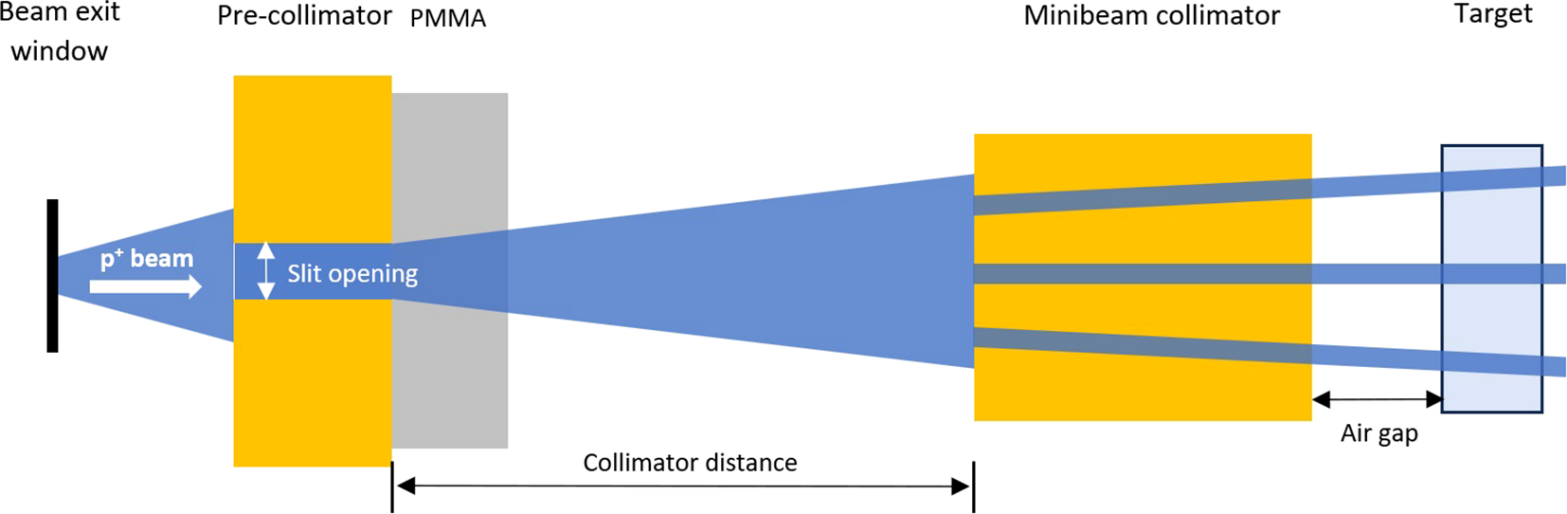
Schematic view (top view) of the minibeam collimation system consisting of a pre-collimator, a PMMA block, and the minibeam collimator.

The minibeam collimation system consisted of a pre-collimator, a polymethylmethacrylate (PMMA) block, and the minibeam collimator itself. Both collimators were made of brass. The components were arranged as shown in **Figure 1**. Monte Carlo simulations were carried out to examine the effect of these components on the resulting minibeam field and to optimize parameters such as the slit opening of the pre-collimator, the thickness of the PMMA block, and the collimator distance. The goal of the optimization was to maximize the PVDR and the time required for dose delivery for minibeams with a width of 250µm, and a ctc of 1000µm. Given that a minimal air gap between minibeam collimator and target is desirable to maximize the PVDR (15), a reasonable distance of 10mm was chosen. The simulations were performed using TOPAS (Version 3.6) (24), employing a physics list composed of the following modules: “g4em-standard_opt3”, “g4h-phy_QGSP_BIC_HP”, “g4h-elastic_HP”, “g4decay”, “g4stopping”, “g4ion-binarycascade” and “g4radioactivedecay”. A range cut of 50µm was applied to all particles. The initial beam was implemented to the simulation assuming a Gaussian shape. The collimation system was developed assuming the 150MeV beam in Dresden. The dose deposition was scored in a 2×2×2cm^3^ PMMA phantom with a spatial resolution of 50µm along the direction of the minibeam pattern (lateral to the beam direction). Additionally, phase space data was collected at various positions within the setup to evaluate the influence of each component.

### 2.3 Proton minibeam dosimetry

Dosimetry for proton minibeams presents particular challenges due to the need for high spatial resolution and quenching effects in the vicinity of the Bragg Peak (25). One established approach that offers sufficient spatial resolution is the use of radiochromic films. However, a drawback is the time of at least 24 hours between exposure and read-out, which is required for the films to stabilize their darkening process (26). Therefore, the microDiamond detector was investigated for its feasibility to provide online dosimetric results for proton minibeams.

#### 2.3.1 Film dosimetry

Radiochromic films change color when exposed to ionizing radiation, providing a measure of absorbed dose. In this experiment, we employed Gafchromic EBT3 films (Ashland, Wilmington, USA). To provide absolute dose values, the films were calibrated with doses ranging from 0-10Gy in a homogeneous proton beam with an energy of 150MeV in Dresden and an energy of 50.5MeV in Seattle. The dose for film calibration was determined with a Markus Chamber (Type: 34045, PTW Freiburg GmbH, Freiburg, Germany) at the irradiation site at both facilities. While the chamber was read out using a Unidos electrometer by PTW in Dresden, a Keithley 6517B electrometer was utilized in Seattle. After irradiation, the films were scanned using a slide scanner (ProScan 10T, reflecta GmbH, Eutingen im Gäu, Germany) with a nominal spatial resolution of 10000DPI. The pixel values were then extracted, and the dose was fitted with the following function (27, 28):


(1) $$D\left(pv\right)=a+\frac{b}{pv-c}$$

where *D* is the dose, *pv* the pixel value and *a*, *b* and *c* the fit parameters.

The dose was acquired by placing the EBT3 film inside a cuboid PMMA phantom (70×60×15mm^3^) at a depth of 5mm. The films were typically cut in 2×2cm^2^ squares and placed parallel to the large plane of the phantom, which was oriented perpendicular to the beam direction. Since the dynamic range of the films is not sufficient to cover both the peak and valley regions simultaneously, two different films were irradiated, aiming for the peak and the valley dose to be around 5Gy in their respective films. The final dose pattern was obtained by overlapping the dose profiles from both films and merging them together. The profiles were normalized to the beam output, measured by the monitor chamber in Dresden or the microDiamond in Seattle, and stitched together at a point of overlapping dose. All films were scanned 48 hours post-irradiation to await darkening. Further details regarding film dosimetry can be obtained from (27).

#### 2.3.2 Microdiamond dosimetry

The microDiamond (Type: 60019, PTW, Freiburg, Germany) is a synthetic single-crystal diamond arranged as a Schottky diode and operated with 0V bias voltage. Its active volume is situated at a depth of 1mm water equivalent from the tip of the cylindrical detector, with a radius of 1.1mm and a thickness of 1µm (29). When operated in the edge-on mode, with the thin side of the sensitive volume facing the beam, the spatial resolution is sufficient to detect minibeams. The microDiamond was positioned inside a PMMA phantom (70×60×15mm^3^) so that its center was placed at a depth of 5mm. The entire phantom was connected to a motorized motion stage that allowed to step through the minibeam field on a micrometer scale. The current produced inside the active volume of the microDiamond by the incident radiation was read out with an electrometer. The step size needed to be small enough to resolve the minibeam pattern yet large enough to maintain a reasonable time of profile measurement. In Dresden, the phantom with the microDiamond was mounted on an array of linear translation stages (Type: LTM 80-75-HSM, OWIS, Staufen, Germany), which enables precise positioning in all three spatial coordinates, whereas in Seattle, the phantom was screwed onto a mecademic robot arm (Mecha500, Mecademic Industrial Robotics, Montreal, Canada). At both facilities, the microDiamond was stepped through the minibeam field with a step size of 20µm. The detected current was converted to a dose rate using a calibration factor that had been determined beforehand through cross-calibration of the microDiamond against a calibrated ionization chamber in a homogeneous proton field. At both facilities, the reference dosimetry was conducted using a Markus chamber (Type: 34045, PTW Freiburg GmbH, Freiburg, Germany). In Dresden, the microDiamond was cross-calibrated with a 150MeV proton beam yielding a calibration factor of $0.92\frac{\text{Gy}}{\text{nC}}$ , whereas in Seattle, the calibration process was carried out with a 50.5MeV proton beam giving a calibration factor of $0.82\frac{\text{Gy}}{\text{nC}}$ . At both facilities, the microDiamond detectors were irradiated with doses ranging from 2-7.5 Gy for calibration. Note that despite being from the same type (TN 60019), two different microDiamond detectors were used in Dresden and Seattle, respectively, which explains the different calibration factors.

## 3 Results

### 3.1 The proton minibeam collimation system

All simulation results in this section assumed the 150MeV beam in Dresden. To evaluate the proton flux through the entire minibeam apparatus, the number of protons reaching the surface of the pre-collimator, the minibeam collimator, and those passing through it was quantified. As depicted in **Figure 1**, the proton beam first encounters a 3cm thick brass pre-collimator with a slit opening of 4mm. Only 47% of the initial protons pass through the pre-collimation before reaching the PMMA block. The minibeam collimator was made of a 5cm thick brass block with 11 slits produced by wire cutting. Each slit had a thickness of 250µm, with a ctc of 1mm at the beam-exit surface, resulting in a total field size of 10×10mm^2^. The slits of the collimator are tilted to align with the divergence of the incoming beam. Assuming a collimator distance of 1m, 24% of the initial protons reach the beam entrance surface of the minibeam collimator, with only 0.5% being able to pass through it.

**Figure 2:**
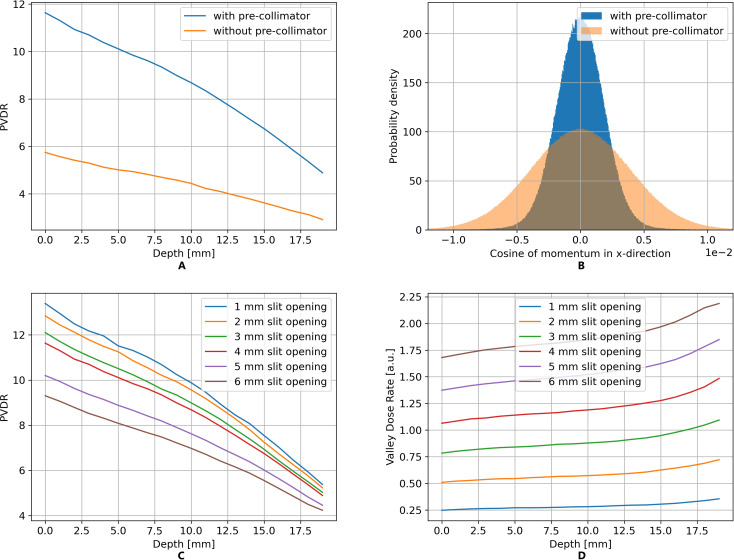
TOPAS simulation results show that the use of a pre-collimator significantly increases the PVDR **(A)** by selectively filtering out protons with higher divergence **(B)**. Further, the PVDR can be adjusted by varying the slit opening **(C)**, leading to a simultaneous change in the valley dose rate **(D)**.

The application of pre-collimation significantly enhances the PVDR of the resulting minibeam field, as shown in **Figure 2A**. The improvement can be attributed to the effect shown in **Figure 2B** displaying the distribution of the direction cosines of the protons in the direction of the minibeam pattern (lateral to the beam direction) entering the central slit of the minibeam collimator. The pre-collimator selectively filters out protons with large directional deviations from the beam axis, which would otherwise primarily blur the valley region. Further, the PVDR can be adjusted by varying the slit opening of the pre-collimator as shown in **Figure 2C**. While a narrower slit increases the PVDR, it simultaneously leads to a drop in the valley dose rate, as apparent from **Figure 2D**. Therefore, the selected slit opening of the primary collimator needs to be a value based on the balance between PVDR and the time required for dose delivery. Therefore, in our experiments, a slit opening of 4mm was chosen.

**Figure 3:**
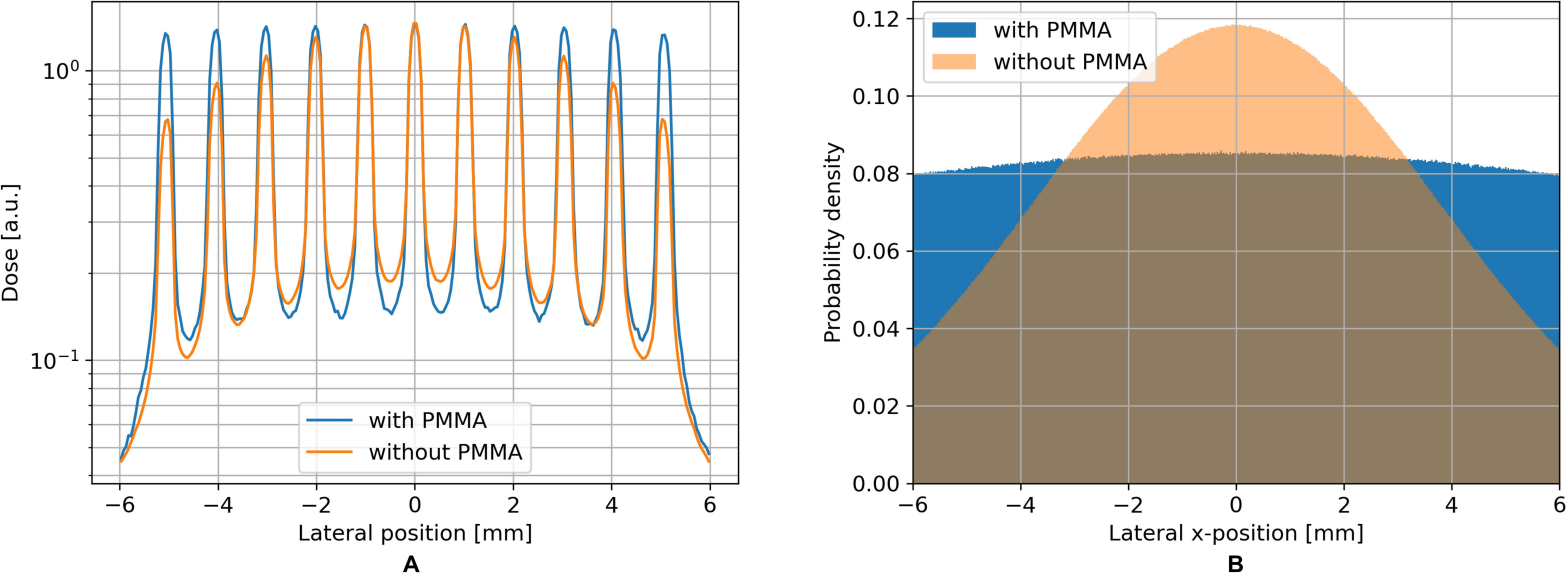
The lateral homogeneity of individual minibeams is ensured by the PMMA block **(A)**, which leads to a more uniform distribution of protons at the collimator surface **(B)**. These results were retrieved by TOPAS simulations.

The absence of the PMMA block behind the pre-collimator results in a decrease in the dose of minibeams further away from the center, as shown in **Figure 3A** (both profiles were normalized to the central peak to compare the lateral dose). In contrast, the inclusion of the PMMA block leads to a more uniform proton distribution at the minibeam collimator as illustrated in **Figure 3B** and thereby ensures that each minibeam throughout the entire field delivers the same dose. The thickness of the PMMA depends on the beam energy and is determined with TOPAS simulations. For the 150MeV beam in Dresden a thickness of 4cm was found to be effective, whereas for the 50.5MeV beam in Seattle a thickness of 0.5cm was chosen.

**Figure 4:**
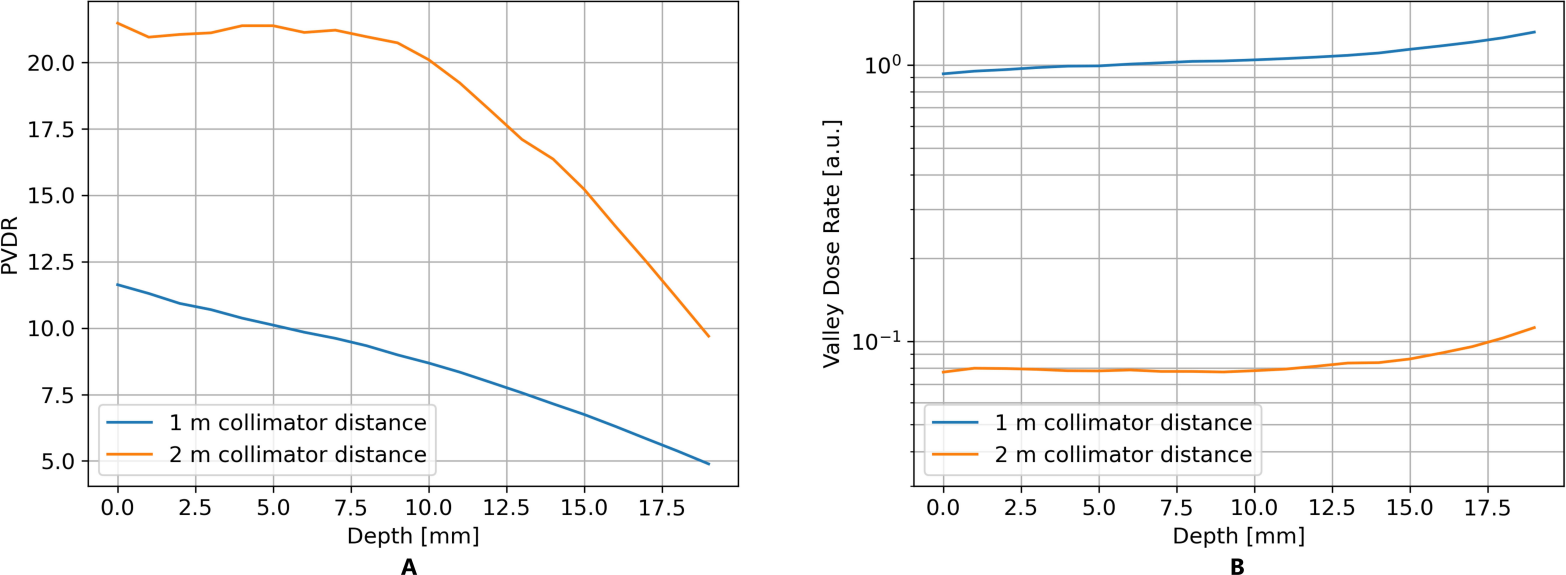
TOPAS simulations showed that the PVDR of the minibeam field can be significantly increased by increasing the collimator distance **(A)**, simultaneously leading to a significant drop in dose rate **(B)**.

Lastly, the PVDR is strongly influenced by the collimator distance. As shown in **Figure 4A**, increasing the collimator distance from 1m to 2m almost doubles the PVDR in the entrance region of the phantom. However, this leads to a reduction in the dose rate in the valley by one order of magnitude, as indicated in **Figure 4B**. Therefore, we decided to proceed with a collimator distance of 1m.

In summary, the minibeam generation system is designed to selectively eliminate particles from the phase space of the initial proton beam that would otherwise compromise the spatial modulation of the resulting minibeam field. In the presented approach, this can be achieved by either narrowing the pre-collimator slit opening or increasing the collimator distance, both of which eliminate protons with a divergence that significantly deviates from the divergence of the minibeam collimator slits. Consequently, this thinning of the phase space results in a decreased dose rate. Therefore, finding a balance between the achievable PVDR and the dose rate is essential.

### 3.2 Implementation and alignment of the minibeam system

**Figure 5:**
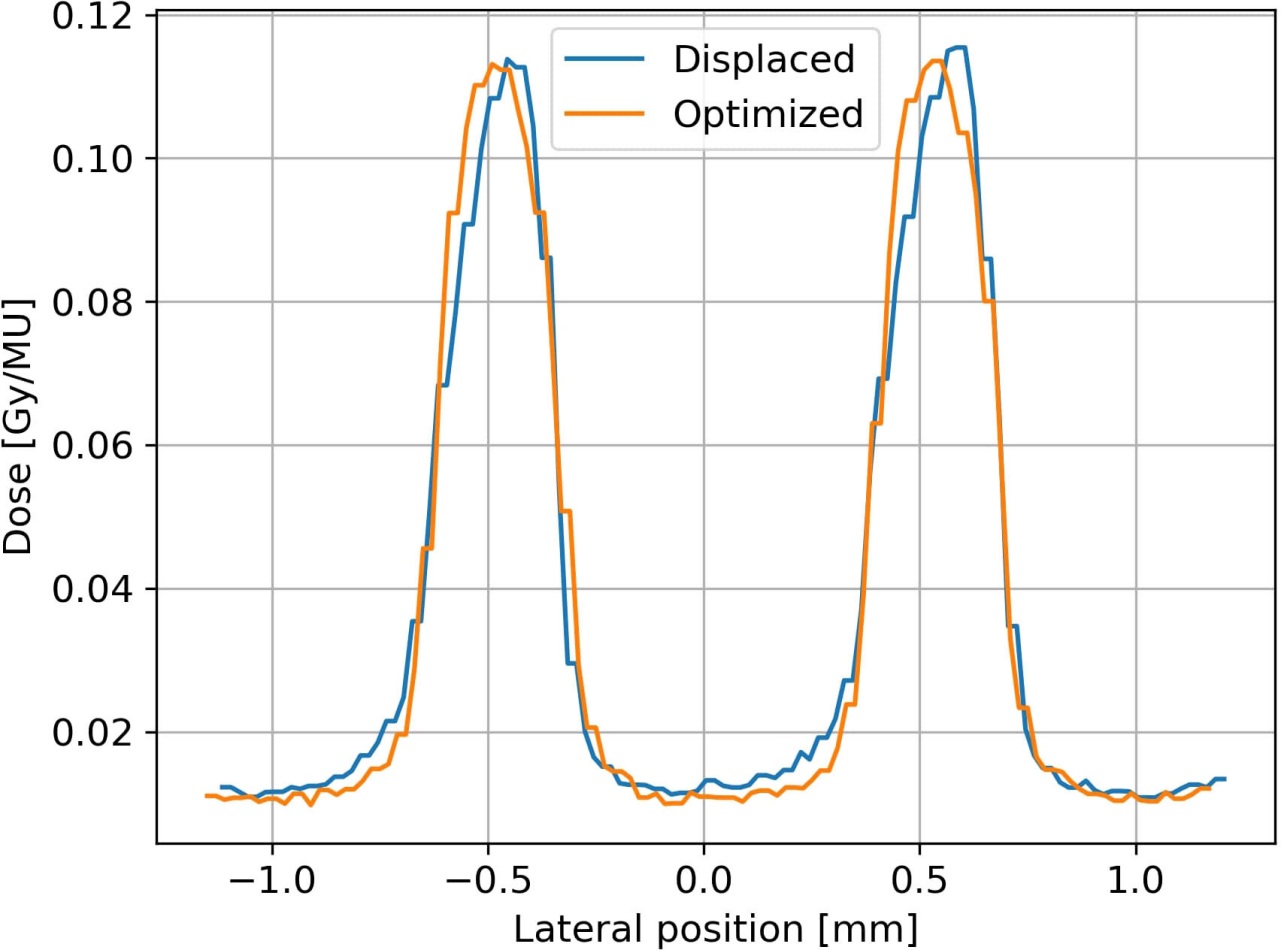
The first microDiamond measurement of the minibeam field after alignment of all components showed an asymmetry of the beams (‘displaced’), which was corrected for by adjusting the angle of the minibeam collimator by 0.09° (‘optimized’).

Pre-collimator and minibeam collimator are both placed on rotational stages (Type: DMT 65-D25-HSM, OWIS, Staufen, Germany) as they are very sensitive to rotational miss-alignments. In the alignment process, each component was sequentially inserted, and the beam intensity through all components was measured with a fluorescent detector positioned 1.2m from the beam exit. While in Dresden, the Lynx detector (IBA, Louvain-La-Neuve, Belgium) was used to detect the beam intensity, the system in Seattle utilized a fluorescent sheet read out by a webcam (Logitech international S.A., Apples, Swiss). The pre-collimator was placed in close vicinity to the beam exit window, and the slit opening was set to 4mm. Its lateral position and height were optimized according to the beam, guided by an installed room laser system. Subsequently, the angle of the pre-collimator was iteratively changed until the measured beam intensity was maximized. The intensity was found to be sensitive to beam angle variations of 0.2°. Next, the PMMA block was put behind the pre-collimator, and the minibeam collimator was inserted into the beam path in 1m distance. The lateral horizontal and lateral vertical position of the minibeam collimator to the beam was again determined in accordance with the lasers visualizing the central beam axis. After optimizing the angular alignment of the collimator, the resulting lateral profile of the minibeams was acquired with the microDiamond. An illustrative example is shown in **Figure 5**, demonstrating that asymmetries in the minibeam profile could be detected with the microDiamond and subsequently corrected by adjusting the collimator angle by 0.09°. The minibeam symmetry was found to be sensitive to angular rotations down to 0.02°. Translational precision of 1mm was required, while accuracy in collimator distance of a few millimeters was sufficient.

### 3.3 Dosimetry of the minibeam field

**Figure 6:**
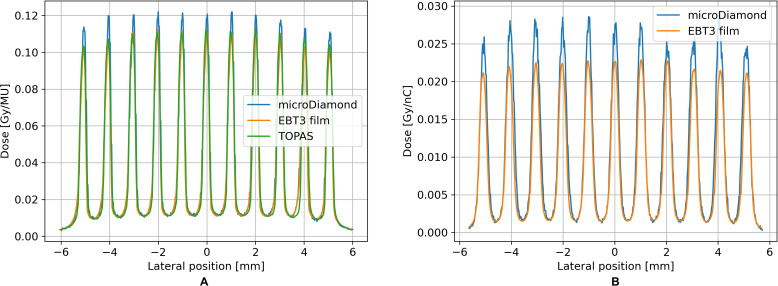
Comparison of dose profiles measured in Dresden (150MeV beam) **(A)** and Seattle (50.5MeV beam) **(B)** displays the comparison of the minibeam dose profiles obtained with film dosimetry and the microDiamond detector using the setup in Seattle. A PVDR of 14 was achieved according to film dosimetry readings.

All dose measurements were conducted with the phantom kept at a constant distance of 10mm from the minibeam collimator exit (‘Air gap’ in **Figure 1**), and a fixed measurement depth of 5mm in PMMA. **Figure 6A** compares the resulting dose distribution obtained with film dosimetry, microDiamond detector, and TOPAS simulation using the setup in Dresden. The valley dose and the transition region between the peak and valley show an excellent agreement with a relative difference of less than 1% between microDiamond and film measurement. The peak dose, however, is overestimated by the microDiamond by 9.5% relative to the film. The TOPAS result was normalized to the valley doses, as these matched for both the film and the microDiamond. The dose profile by TOPAS showed a near-perfect match with the EBT3 film reading. With our setup in Dresden, we accomplished a PVDR of 10 at 5mm depth in a PMMA phantom placed 10mm from the collimator. The minibeams exhibited a full width at half maximum (FHWM) of 265µm with a ctc of 1020µm.

**Figure 6B** displays the comparison of the minibeam dose profiles obtained with film dosimetry and the microDiamond detector using the setup in Seattle. A PVDR of 14 was achieved according to film dosimetry readings. The agreement between microdiamond and EBT3 film was once again excellent in the valley region with a deviation of below 1%. In contrast, the peak dose was overestimated by 21% by the microDiamond. The FWHM measured with the films was 320µm and the ctc 1020µm. No comparison with TOPAS simulation was made for the Seattle beamline. A valley dose rate of $3\frac{\text{Gy}}{\text{min}}$ and $4.3\frac{\text{Gy}}{\text{min}}$ was achieved in Seattle and Dresden, respectively.

## 4 Discussion

In this study, we successfully optimized a system to produce proton minibeams. Using a 150MeV proton beam, we achieved a PVDR of 10 at 5mm phantom depth with an air gap of 10mm between the phantom and the minibeam collimator, which is reasonable for *in vitro* and *in vivo* experiments. Other proton minibeam setups using comparable proton energies report PVDRs of 5 (19), 9 (20) and 11.3 (21), all at 0cm phantom depth. The primary distinction between our setup and others lies in the ctc to beam width ratio (CBR). While other setups used a beam width of 400µm and ctc ranging from 2.8mm to 4mm resulting in a CBR of 7-10, our minibeams are smaller with a CBR of 4. We decided to use smaller minibeams due to the small target size when treating mouse organs. Although generally, the PVDR increases with higher CBR (6), our setup compares well with the others despite producing smaller minibeams. Consequently, to the best of our knowledge, we present the first setup that is capable of producing small high-energy proton minibeams while still maintaining a PVDR comparable to other setups. Having achieved a proton minibeam quality comparable to others, we aim to perform orthotopic mouse irradiations targeting the brain and the lung. We plan to investigate the tumor response, side effects and radiobiological mechanisms of pMBRT.

With the 50.5MeV beam in Seattle, our setup was able to produce minibeams with a PVDR of 14 in 5mm phantom depth again with an air-gap of 10mm. The only other setup using the same beam energy reported a PVDR of 10 at 5mm phantom depth (16).

Furthermore, we characterized our setup at both beamlines with film dosimetry and investigated the usability of a microDiamond detector for proton minibeam dosimetry. We established a calibration protocol for the microDiamond and successfully used it to measure the minibeam profile. For both beamlines, we found excellent agreement between microDiamond and EBT3 films in the valley region. For the peak dose, however, we observed a discrepancy between the microDiamond and film readings of around 10% and 20% for 150MeV and 50.5MeV minibeams, respectively, resulting in an increased PVDR measured by the diamond detector. Since the PVDR given by the TOPAS simulation agrees with the film measurement, this discrepancy is attributed to the microDiamond’s differential response depending on whether its active volume is located in the peak or valley of the dose distribution. This behavior has previously been reported by Sotiropoulos et al. (30), who modeled the microDiamond in a TOPAS simulation and compared the dose depositions in the detector to the dose deposited in water. They found that the dose-response of the microDiamond is dependent on the position of the active volume within the minibeam field, necessitating position-specific radiation correction factors. Specifically, they reported that for 100MeV and 160MeV proton minibeams, a radiation correction factor of approximately 0.9 is needed when the active volume is in the peak, while in the valley, the correction factor is 1 (30). These findings by Sotiropoulos et al. align perfectly with our data from Dresden. However, correction factors for 50.5MeV proton minibeams have not yet been investigated. The excellent match in valley dose between microDiamond and films suggests that the discrepancy in the peak might be subject to a similar effect. However, further experiments investigating the energy dependence of this effect and finding the threshold in beam width by iteratively reducing the beam size until the effect appears should provide a better understanding of the response of the microDiamond in proton minibeams.

Clinical translation of pMBRT faces two key questions: First is the available technology sufficient to generate proton minibeams that are suitable to treat a human? Second, is the superiority of pMBRT compared to conventional treatment sufficiently understood or proven? In our study, we showed that a significant drawback of using collimators is the limited PVDRs and dose rates achievable, since they will always be compromised by scattered particles caused by the collimators. The only way to increase the PVDR further is to increase the thickness of the collimator, which will further limit the achievable dose rate. In our setup, 99.5% of initial protons are lost in the selection processes. Therefore, the use of collimated proton minibeams may not be ideal in a clinical environment, and possibilities of generating minibeams using magnetic focussing are being investigated that might be able to mitigate some of the limitations posed by collimators. An idea for a clinical pMBRT nozzle has been proposed by Schneider et al. (14), and a facility with magnetically scanned proton minibeam irradiation for pre-clinical research is currently being developed by Reindl et al. (31). However, due to substantial technical demands and the quality of the beam being fed into the nozzle, these approaches have not yet been realized.

Further, either the underlying biological mechanisms of pMBRT have to be understood, or an ideal set of minibeam field parameters (FWHM of the minibeams, ctc, PVDR) has to be identified that proves to outperform conventional treatment before treating humans with pMBRT. Here, our setup, which is flexible, transportable, and usable at any proton source, can aid standardized pre-clinical studies, which are key to answering the open questions.

To replicate this setup at any proton source, we would advise to perform Monte Carlo simulations using the phase space of the respective source. While core components and their arrangement remain unchanged, parameters such as the opening of the pre-collimator and the energy-dependent PMMA plate will need to be optimized according to the phase space of the proton facility.

## 5 Conclusion

In conclusion, our study represents an advancement in minibeam generation through mechanical collimation. To the best of our knowledge, our setup is unique in its ability to produce 250µm wide minibeams with a ctc of 1000µm at a proton energy of 150MeV, while still achieving a PVDR as high as 10 in a practical experimental geometry. We demonstrated the portability and independence of specific beamlines of our setup by successfully using our system at two different beamlines at different proton beam energies. Additionally, we characterized and validated our setup at both beamlines with film dosimetry and investigated the microDiamond as an online alternative to film dosimetry for dose measurement. Our experimental results show the need for position-specific correction factors when using the microDiamond to determine the dose deposition by minibeams. With these developments, we are ready to perform both *in vitro* and *in vivo* (small animal) experiments using proton minibeams.

## Data Availability

The original contributions presented in the study are included in the article/supplementary material. Further inquiries can be directed to the corresponding author.
